# First Line Treatment Response in Patients with Transmitted HIV Drug Resistance and Well Defined Time Point of HIV Infection: Updated Results from the German HIV-1 Seroconverter Study

**DOI:** 10.1371/journal.pone.0095956

**Published:** 2014-05-01

**Authors:** Fabia zu Knyphausen, Ramona Scheufele, Claudia Kücherer, Klaus Jansen, Sybille Somogyi, Stephan Dupke, Heiko Jessen, Dirk Schürmann, Osamah Hamouda, Karolin Meixenberger, Barbara Bartmeyer

**Affiliations:** 1 Department of Nephrology, Charité, University Medicine, Berlin, Germany; 2 Department of Infectious Disease Epidemiology, HIV/AIDS, STI and Blood Born Infections, Robert Koch Institute, Berlin, Germany; 3 Department of Infectious Diseases, HIV and Other Retroviruses, Robert Koch Institute, Berlin, Germany; 4 Medical Care Centre Driesener Strasse, Berlin, Germany; 5 Medical Care Centre Jessen, Berlin, Germany; 6 Department of Infectious Diseases and Pulmonary Medicine, Charité, University Medicine, Berlin, Germany; University of Athens, Medical School, Greece

## Abstract

**Background:**

Transmission of drug-resistant HIV-1 (TDR) can impair the virologic response to antiretroviral combination therapy. Aim of the study was to assess the impact of TDR on treatment success of resistance test-guided first-line therapy in the German HIV-1 Seroconverter Cohort for patients infected with HIV between 1996 and 2010. An update of the prevalence of TDR and trend over time was performed.

**Methods:**

Data of 1,667 HIV-infected individuals who seroconverted between 1996 and 2010 were analysed. The WHO drug resistance mutations list was used to identify resistance-associated HIV mutations in drug-naïve patients for epidemiological analysis. For treatment success analysis the Stanford algorithm was used to classify a subset of 323 drug-naïve genotyped patients who received a first-line cART into three resistance groups: patients without TDR, patients with TDR and fully active cART and patients with TDR and non-fully active cART. The frequency of virologic failure 5 to 12 months after treatment initiation was determined.

**Results:**

Prevalence of TDR was stable at a high mean level of 11.9% (198/1,667) in the HIV-1 Seroconverter Cohort without significant trend over time. Nucleotide reverse transcriptase inhibitor resistance was predominant (6.0%) and decreased significantly over time (OR = 0.92, CI = 0.87–0.98, p = 0.01). Non-nucleoside reverse transcriptase inhibitor (2.4%; OR = 1.00, CI = 0.92–1.09, p = 0.96) and protease inhibitor resistance (2.0%; OR = 0.94, CI = 0.861.03, p = 0.17) remained stable. Virologic failure was observed in 6.5% of patients with TDR receiving fully active cART, 5,6% of patients with TDR receiving non-fully active cART and 3.2% of patients without TDR. The difference between the three groups was not significant (p = 0.41).

**Conclusion:**

Overall prevalence of TDR remained stable at a rather high level. No significant differences in the frequency of virologic failure were identified during first-line cART between patients with TDR and fully-active cART, patients with TDR and non-fully active cART and patients without TDR.

## Introduction

The wide use of combination antiretroviral therapy (cART) succeeded in sustained inhibition of viral replication and reduced significantly the morbidity and mortality of HIV disease [1]–[4]. However, treatment options can be impaired by the development of antiretroviral drug resistance. Insufficient virus suppression during cART is the main factor for selection of resistant HIV-1 variants. Resistant virus strains can be transmitted to new hosts and, subsequently, can lead to antiretroviral treatment failure [5]. Loss of efficacy of cART would have extensive consequences as the containment of disease is nearly exclusively accredited to effective therapy. Estimates of the prevalence of transmitted drug resistance (TDR) in Europe range from 3.3% to 14.2% [6]–[31] with stable [6], [20], [25], [26], [31] or decreasing [7], [10], [21], [24], [27]–[30] trends over time. However, the increasing global use of antiretroviral drugs may in turn increase the number of patients at risk to select resistant viral variants under incomplete cART and may concomitantly raise the risk of TDR.

The prevalence of people living with HIV in Germany has been increasing continuously, and concomitantly the proportion of patients treated with antiretrovirals has been increasing [32]. Moreover, despite the high proportion of treated HIV patients in clinical care, current estimates show an increase in the proportion of individuals newly infected with HIV but still undiagnosed [32]. An increase of both the use of cART and of patients recently infected with HIV with unsuppressed viraemia raises the risk of TDR in Germany. Hence, the surveillance of the prevalence and time trends of TDR, resistance testing as clinical practice and analyses of treatment success in patients with TDR receiving first-line cART are of great importance.

Numerous studies demonstrated a significantly higher rate of virologic failure in subjects with TDR if the antiretroviral regimen comprised at least one drug showing reduced activity [33]–[36]. However, some controversial data exist regarding the impact of TDR to treatment response if first-line treatment was resistance test guided. At least in studies with short duration of observation a comparable efficacy of first-line cART was observed in patients with and without TDR if regimens comprised only active drugs [8], [15], [37]. Other studies found a higher proportion of virologic failure in the participants with TDR although they were receiving fully active therapy [34], [38]. In particular, higher odds ratio (OR) for failure was determined if a non-nucleoside reverse transcriptase inhibitor comprising regimen was administered [34]. In patients from the German HIV-1 Seroconverter Study infected between 1996 and 2007 no difference was observed in response to first-line cART between patients with and without TDR [39]. The aim of our study was to update the analysis of prevalence and trend of TDR in the German HIV-1 Seroconverter Cohort between 1996 and 2010 and to evaluate the impact of TDR on first-line treatment success within the first year of treatment. In addition, cART prescription practice and risk factors associated with TDR were analysed.

## Methods

### Study Design

The German HIV-1 Seroconverter Study received ethical approval first in 2005 by the ethic committee of the Charité, University Medicine Berlin. Ethical approval was amended in December 2012 and the amendment was confirmed by the committee in January 2013. Patients have to sign written informed consent. The HIV-1 Seroconverter Study is a nationwide multicentre open prospective cohort study which includes HIV-1-infected individuals for whom the date of HIV-1 seroconversion is known or reliably estimated by laboratory diagnostics. All patients enrolled in the study signed an informed consent form. Twenty-two clinics, forty private medical practices specialized in the care of HIV patients and seven public health offices are involved in the recruitment of patients. Epidemiologic, clinical and laboratory data are collected on a yearly basis using a standardised questionnaire. Inclusion criteria are (1) age over 18 years and (2) an acute seroconversion confirmed by laboratory diagnostics (acute HIV-1 seroconverters) or a documented HIV-1 seroconversion (documented HIV-1 seroconverters) with at most a 3-year interval between the last negative and the first confirmed positive HIV antibody test.

Acute HIV-1 seroconverters are defined by the following laboratory diagnostic criteria: (1) detectable HIV-1 RNA or p24 antigen combined with a negative or indeterminate ELISA result or (2) reactive HIV-1 ELISA combined with a negative or indeterminate immunoblot result with confirmation of complete seroconversion within six months.

The blood sampling date of the first reactive test (acute seroconverters) or the arithmetic mean between the last negative and the first confirmed positive HIV antibody test (documented seroconverter) are considered as date of infection.

### Genotypic Resistance Testing

Blood samples from each individual were collected at date of enrolment and transported to the Robert Koch Institute within 48 hours after venipuncture. 81.3% (1355/1667) of the blood samples were taken within 365 days after calculated date of infection. Genotypic resistance testing was performed for drug-naïve patients. The methods used have been described previously [18], [40]. In brief, the ViroSeq HIV-1 Genotyping System (Abbott, Wiesbaden, Germany) was used to determine genotypic resistance. Alternatively, an in-house *pol* RT-PCR system [41] was used to amplify a 1.5 kb *pol* fragment encoding the complete protease (99 amino acids) and reverse transcriptase (1–296 amino acids). Nucleotide sequences from drug-naïve patients were processed through the Stanford Genotypic Resistance Interpretation Algorithm (HIVSeqProgram, version 6.1.1 at http://hivdb.stanford.edu/). The identified resistance mutations were analysed by using both the WHO surveillance drug resistance mutations list (SDRM) [42] and the Stanford algorithm. The Stanford algorithm comprises the mutations contained in the IAS-USA drug resistance mutation list. The presence of at least one of the mutations according to the SDRM list was considered as TDR for epidemiological analysis. Resistance mutations conferring low, intermediate and high levels of predicted resistance according to the Stanford algorithm were considered as TDR for the analysis of first-line cARTand clinical context. As a consequence of the use of the Stanford algorithm HIV-1 strains encoding revertant substitutions of the T215Y/F in the reverse transcriptase causing high level resistance to thymidine analogues are included in the treatment response analyses because they are scored with low level resistance to AZT and D4T, and with potential level of cross resistance to ABC, DDI and TDF. The revertants are not contained in the IAS 2013 list of drug resistance mutations [43].

### Prevalence of TDR

The prevalence of TDR was calculated as the frequency of TDR in all drug-naïve seroconverters genotyped by year of HIV-1 seroconversion (N = 1,667; 1996–2010) and by drug classes (nucleotide reverse transcriptase inhibitors [NRTIs], non-nucleoside reverse transcriptase inhibitors [NNRTIs], protease inhibitors [PIs]). To calculate the prevalence of each of the drug classes, occurrence of drug class resistance was cumulated from mono-, dual- and triple-resistant variants per year of seroconversion.

HIV subtype was assigned using the REGA tool [44] (REGA HIV-1 & 2 Automated Subtyping Tool Version 2.0; http://www.bioafrica.net/rega-genotype/html/subtypinghiv.html). In case the REGA tool could not assign a subtype, the distance-based neighbour joining phylogenetic tree (PHYLIP version 3.6; J. Felsenstein) was calculated using an extended panel of subtype reference sequences (n = 159) from the HIV sequence data base (http://www.hiv.lanl.gov/content/sequence/NEWALIGN/align.html#ref).

### Analysis of Treatment Success

For analyses of the effect of TDR on treatment success, only those patients were included (i) who were HIV-genotyped while drug-naïve, (ii) who received a first-line cART for a minimum of five months, (iii) whose CD4-cell count and HIV RNA viral load (VL) were determined within 90 days before start of therapy, and (iv) who had at least two consecutive VL measurements 5–12 months after initiation of first-line cART or until the end of first-line cART in case first-line cART treatment duration was shorter than 12 months (N = 323).

Patients were classified into three groups based on the prediction of resistance by the Stanford algorithm: 1) patients without TDR [no resistance or potential low-level resistance mutation (Stanford level 1 and 2)], 2) patients with TDR receiving fully active first-line cART [at least one low-level and/or intermediate and/or high-level resistance mutation (Stanford levels 3–5) without affecting the prescribed first-line cART], 3) patients with TDR and non-fully active first-line cART [at least one low-level and/or intermediate and/or high level resistance mutation (Stanford level 3–5) affecting at least one of the prescribed drugs of their first-line cART].

The primary outcome of this investigation was the analysis of the frequency of virologic failure in patients without TDR, patients with TDR receiving fully active first-line cART and patients with TDR receiving non-fully active first-line cART. Since VLs were determined by using various commercial kits with different detection limits during the long observation time of the study, VLs that remained permanently below the detection limit of 500 copies/ml were considered as treatment success. A transient viraemia between 500–1,000 copies/ml with a subsequent drop of VL below 500 copies/ml was considered as a blip and also assessed as treatment success. Virologic failure was defined to be the case if at least one of the VL measurements was above 1,000 copies/ml or if two consecutive VLs were above 500 copies/ml.

### Statistical Analysis

Descriptive statistics for continuous variables were calculated as medians and interquartile ranges (IQR). The Mann-Whitney-U test (MWT) and the Kruskal-Wallis test were used to compare differences between groups. Proportions were given with a 95%-Wilson score confidence interval (CI) based on binomial distribution. Differences in proportions were assessed by Fisher’s exact test and chi-squared test.

For time-to-event analyses, Kaplan-Meier analyses and log rank statistics were used in order to derive differences in duration of first-line cART and the time interval between HIV-1 seroconversion and first-line cART. Univariable and multivariable cox proportional hazard model was performed to compare time-to-event analyses of more than two resistance groups. For treatment success analysis univariable and multivariable logistic regression was used to compare differences in the frequency of virologic failure between resistance groups. Univariable and multivariable logistic regression models were calculated to determine predictors of TDR such as subtype, age, sex and exposure to cART. Trend analysis in the prevalence of TDR was performed by using multivariable logistic regression. Multivariable models were adjusted for sex, age and transmission group. A two-sided p-value below 0.05 was considered significant in all statistical tests applied. All data were analysed using STATA 10 (www.stata.com/stata10 **)**.

## Results

### Characteristics of the Study Population

**Figure 1:**
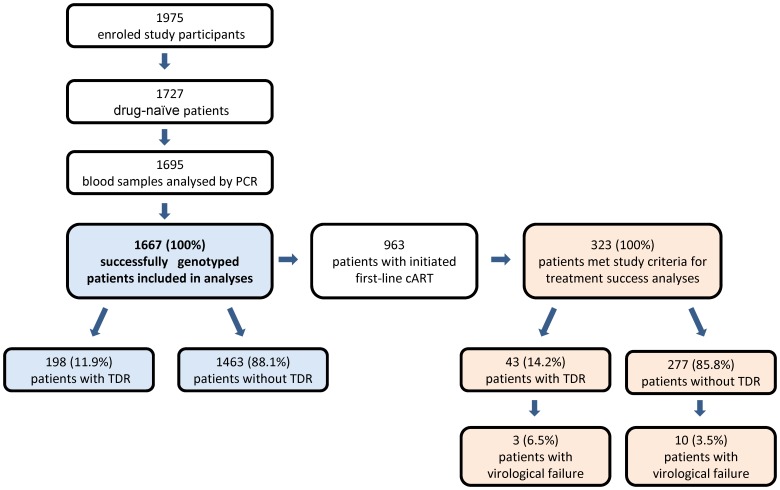
Flow chart of patient groups.

A total of 1,727 blood samples from 1,975 HIV-1-positive patients with known or estimated date of seroconversion were available for analysis. Genotyping was performed in 1,695/1,727 (98.2%) of the blood samples available. 1,667 HIV-infected drug-naïve patients with a successful genotypic resistance test were included in the analysis (figure 1). Included study participants seroconverted between 1 January 1996 and 10 November 2010.

According to the criteria defined above, 44.8% of patients were acute HIV-1 seroconverters and 55.2% of patients documented HIV-1 seroconverters. At date of seroconversion, most patients (93.5%) were classified as CDC category A. Most patients were male (95.3%) and the median age was 33 years (IQR 27–39). The predominant route of HIV transmission was sex between men (MSM; 87.9%) and the dominant HIV-1 subtype was B (92.3%) (table 1).

**Table 1:** Characteristics of study population.

	Total	Patients with TDR*	Patients without TDR*	OR (95%CI)"	p-value
**Genotypic resistance tested patients (%)**	1,667 (100)	198 (11.9)	1,469 (88.1)		
**Type of seroconversion (%)**					0.49^1^
Acute	746 (44.8)	84 (42.4)	662 (45.1)		
Documented	921 (55.2)	114 (57.6)	807 (54.9)		
**CDC category at sc^a^ (%)**					0.13^1^
A	1,544 (92.6)	181 (91.4)	1,363 (92.8)		
B	94 (5.6)	16 (8.1)	78 (5.3)		
C	29 (1.7)	1 (0.5)	28 (1.9)		
**Sex**				2.06 (0.824–6.57)	0.15^1^
Men (%)	1,588 (95.3)	193 (97.5)	1,395 (95.0)		
Women (%)	78 (4.7)	5 (2.5)	73 (5.0%)		
**Median age at sc (IQR** * **)**	33 (27–39)	34 (26–39)	33 (27–39)		0.87^2^
**Subtype** (n = 1,664)					0.00^1^
B subtype (%)	1,536 (92.3)	195 (99.0)	1,341 (91.4)		
Non-B subtype (%)	128 (7.7)	2 (1.0)	126 (8.6)		
**Transmission group (%)**					
MSM°	1,465 (87.9)	182 (91.9)	1,283 (87.3)	1.65 (0.96–3.01)	0.06^1^
Heterosexual contacts	110 (6.6)	8 (4)	102 (7)	0.56 (0.53–1.18)	0.16^1^
Exposure at work	5 (0.3)	0	5 (0,3)		1.0^1^
High prevalence country	30 (1.8)	0	30 (2)		0.04^1^
Intravenous drug use	21 (1.2)	1 (0.5)	20 (1.4)	0.37 (0.01–2.32)	0.5^1^
Unknown	20 (1.2)	4 (2)	16 (1.1)	1.87 (0.45–5.88)	0.29^1^
**Median VL at sc** (log/ml) (IQR*) (n)	5.25 (4.5–5.9) (785)	5.2 (4.5–6.2) (82)	5.26 (4.5–5.9) (669)		0.67^2^
**Median CD4 cc** ∼ **at sc^a^** (cells/µl) (IQR*) (n)	473 (350–642) (748)	528 (374–647) (79)	470 (348–641) (669)		0.48^2^
**Median duration of FL-ART** × (day) (CI) (n)	810 (571–974) (922)	477 (320–974) (122)	823 (595–1182) (800)		0.045^3^
**Median time to FL-ART** × (day) (CI) (n)	1.011 (928–1.071)	896 (736–1.156)	1.014 (939–1.079)		0.61^3^
**Median CD4 cc** ∼ **at FL-ART** × **start** (cells/µl) (IQR*) (n)	300.5 (213–408) (778)	303 (204–406) (102)	299.5 (214–422) (676)		0.92^2^
**Median VL at FL-ART** × **start** (log/ml) (IQR*) (n)	5.00 (4.5–5.5) (795)	5.11 (4.5–5.6) (108)	5.00 (4.5–5.5) (687)		0.5^2^

^a^sc: seroconversion.

^1^Fisher exact test.

^2^Mann-Whitney-U test.

^3^Log rank test.

°MSM: men who have sex with men].

*IQR: Interquartile range.

"CI: 95% confidence interval.

∼ cc: cell count.

× FL-ART: first-line cART.

### Prevalence of TDR in the HIV-1 Seroconverter Cohort

The overall prevalence of TDR between 1996 and 2010 (year of seroconversion) was 11.9% (CI = 10.3–13.4;198/1667) according to the WHO resistance mutation list (Stanford: 16,1%; CI = 14.3–17.9; 267/1667). Neither univariable (OR = 0.99, CI = 0.99–1.00, p = 0.06) nor multivariable trend analyses adjusted for age, transmission group and sex showed a significant association between date of infection and prevalence of TDR.

Transmitted NRTI resistance was identified most frequently with 6.0% (Stanford: 5.2%) followed by NNRTI resistance with 2.4% (Stanford: 5.2%) and PI resistance 2.0% (Stanford: 2.8%). Dual-class resistance was detected in 1.2% (Stanford: 2.7%) of patients. Triple-class resistance was only observed in 0.3% (Stanford: 0.24%) of patients.

The prevalence of transmitted NRTI resistance according to the SDRM list decreased strongly between 1999 and 2000 and its overall trend was highly significant (OR = 0.92 per calendar year, CI = 0.87–0.98, p = 0.01). Prevalence of NNRTI and PI resistance has remained rather stable over the last years and did not show any significant trend (NNRTI: OR = 1.00, CI = 0.92–1.09, p = 0.96; PI: OR = 0.94, CI = 0.86–1.03, p = 0.17).

### Factors Associated with TDR

The prevalence of TDR according to the SDRM list was highest for MSM (12.4%). There were no significant differences between patients with and without TDR regarding sex, median age, frequency of acute or documented HIV-1 seroconversion, CDC stage, CD4-cell count and VL at HIV-1 seroconversion. In univariable analysis infection with HIV-1 subtype B was highly significantly associated with TDR (OR: 9.2, CI 95%: 2.2–37.3, p = 0.002). This factor was the only independent predictor of TDR also in the multivariable model. Univariable and multivariable analysis showed no association between other variables like sex, age and route of HIV transmission and TDR.

### First-line cART

First-line cART was initiated in 56.1% (936/1,667) of all patients with successful genotypic resistance test. The median time interval between HIV-1 seroconversion and start of first-line cART for all patients was 1,011 days (CI 95% = 928–1,071). The median duration of first-line cART was 810 days (CI 95% = 571–974).

First-line cART was initiated in 55.3% (813/1,469) of patients without TDR and in 62.1% (123/198) of patients with TDR. Acording to the Stanford algorithm 38.2% (47/123) of treated patients with TDR received a first-line regimen which comprised at least one inactive drug. 19/47 patients receiving a non fully-active regimen had a high level drug resistance (Stanford level 5). Kaplan-Meier analysis showed a significant difference in duration of first-line cART when comparing patients with and without TDR (cART duration: 627 vs. 563 days, p = 0.019) (duration: 800 vs. 444 days, p = 0.048). There was no significant statistical difference between patients with and without TDR regarding the time interval between HIV-1 seroconversion and start of first-line cART (1,014 vs. 896 days, p = 0.61) and CD4-cell count (299 vs. 303 cells/µl, p = 0.92) and VL at start of first-line cART (100,000 vs. 129,500 copies/ml, p = 0.5).

Patients without TDR were treated most frequently with a regimen of 2NRTI/1NNRTI (43.3%). With similar frequency a regimen based on 2NRTI/1PIr was used as first-line cART (42.4%). The third most common regimen comprised an integrase inhibitor and two NRTIs (2.8%). Within the different drug classes the most commonly prescribed PI was lopinavir (79.7%), the most common NNRTIs were efavirenz (65.8%) and nevirapine (33.4%). Among NRTIs emtricitabine (64.2%) and tenofovir (65.8%) were most commonly administered.

**Figure 2:**
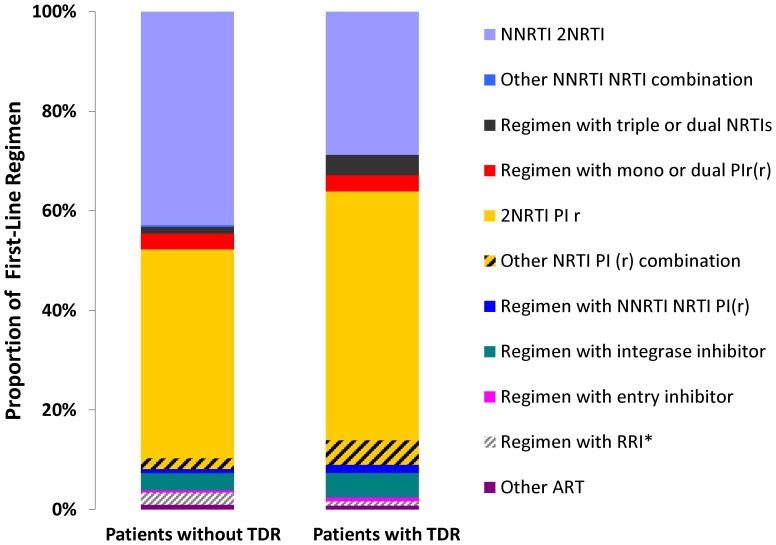
Composition of first-line cART 1996–2010.

Patients with TDR most frequently received a 2NRTI/1PIr containing regimen (49.6%). The combination of 2NRTI/1NNRTI was used for 28.5% of the patients with TDR. Among PIs lopinavir was predominantly prescribed (51.9%). Efavirenz was favoured over nevirapine among NNRTIs (65.8% vs. 28.9%). Emtricitabine and tenofovir were the most commonly administered NRTIs (61.3% and 63%) (table 2 and figure 2).

**Table 2:** Prescribing practices of NRTIs, NNRTIs and PIs in first-line treatment*.

Agents (generic name)	Patients with TDR∧ (%)	Patients without TDR∧ (%)
NRTI		
Zidovudine	18.5	21.8
Lamivudine	33.6	27.9
Abacavir	14.3	9.3
Didanosine	4.2	3.3
Emtricitabine	61.3	64.3
Tenofovir	63.0	65.8
Stavudine	8.4	2.0
NNRTI		
Rilpivirine	0	0.79
Efavirenz	65.8	65.8
Etravirine	0	0
Nevirapine	28.9	33.4
PI		
Tipranavir	3.8	3.1
Saquinavir	8.9	5.8
Atazanavir	12.7	12.8
Lopinavir r	51.9	54.4
Darunavir	12.7	14.3
Fosamprenavir	3.8	3.4
Nelfinavir	5.1	5.6

∧TDR: transmitted drug resistance according to [42].

*Drugs listed also if administered in combined pills.

### Treatment Success Analysis

323 patients met the inclusion criteria for the analysis of treatment success.

According to the Stanford algorithm there were 41 patients with TDR receiving fully active cART, 18 patients with TDR receiving a non-fully active cART and 264 patients without TDR receiving cART. There were no significant differences between the three groups regarding VL measurements at start of cART, sex, age, transmission route and time interval between HIV-1 seroconversion and start of first-line cART (table 3). The CD4-cell count of patients with TDR and non-fully active cART was significantly higher than in patients without TDR or patients with TDR receiving fully active cART (322 vs. 270 vs. 272; p = 0,02).

**Table 3:** Characteristics of patients included in treatment success analysis**.**

	Total	Patients with TDR∧receiving fully activecART	Patients withTDR∧receiving non-fullyactive cART	Patientswithout TDR∧	p-value
Patients of sample (%)	323 (100)	41 (12.7)	18 (5,6)	264 (81.7)	
Sex					0.33^1^
men (%)	311 (96.3)	38 (92,7)	18 (100)	255 (95.7)	
women (%)	12 (3.7)	3 (7,3)	0	9 (4.3)	
Median age at sc^a^ (IQR)*	35 (29–40)	37 (29–43)	33 (29–38)	34 (29–40)	0.43
Transmission group					
MSM° (%)	292 (90.4)	34 (82.9)	16 (88.8)	242 (91.7)	0.20^1^
Heterosexual contacts	23 (7.1)	6 (14.6)	1 (5.6)	16 (6.0)	0.13^1^
High prevalence country	1 (0.3)	0	0	1 (0.4)	0.89^1^
Intravenous drug use	1 (0.3)	1 (2.5)	0	0	0,03^1^
Unknown	20 (1.2)	0	1 (5.6)	5 (1.9)	0.35^1^
Median time to FL-ART = (day) (CI”)	632 (559–684)	757 (557–1,014)	550 (244–800)	613 (530–666)	
Univariable HR^3^ for time to FL-ART = (p-value)		0.87 (0.43)	1.38 (0.19)	1.00	
Multivariable HR for time to FL-ART = (p-value)		0.87 (0.42)	1.44 (0.14)	1.00	
Median VL× at start of FL-ART = (log/ml) (IQR*)	5.3 (4.5–5.9)	5.23 (4.5–5.6)	4.59 (4.3–5.5)	4.95 (4.5–5.4)	0.42^2^
Median CD4 cc**∼** at start of FL-ART = (cells/µl)(IQR*) (n)	277 (200–380)	270 (210–358)	350.5 (290–488)	272 (194.5–377.5)	0.02^2^
Median duration of FL-ART = (day) (CI”)	1,919 (1,542–*)	1,400 (916–*)	1,681 (575–2,112)	2,386 (1,542–*)	
Univariable HR for duration of FL-ART = (p-value)		1.12 (0.72)	1.29 (0.52)	1.00	
Multivariable HR for duration of FL-ART = (p-value)		1.16 (0.63)	1.3 (0.48)	1.00	
Pat. with treatment success (2 cons. VL×<500 copies/ml) (%)	310 (96.0)	43 (93.5)	17 (94.4)	256 (96.4)	
Pat. with virologic failure (min. 1 VL×>500 copies/ml) (%)	13 (4.0)	3 (6.5)	1 (5.6)	9 (3.4)	0.47^1^
Univariable odds ratio to compare virologicalfailure (p-value)		2.24 (0.24)	1.67 (0.64)	1.00	
Multivariable odds ratio to compare virologicalfailure (p-value)		2.34 (0.22)	1.78 (0.60)	1.00	
Pat. with blip (transient VL× <1,000 und>500 copies/ml) (%)	2 (0.6)	0	0	2 (0.8)	

^a^ sc: seroconversion.

^1^ chi-square test.

^2^ Kruskal-wallis test.

^3^ HR: hazard ratio.

° MSM: men who have sex with men.

∧ TDR: transmitted drug resistance according to Stanford algorithm.

* IQR: interquartile range.

“ CI: 95% confidence interval.

∼ cc: cell count.

× VL: Viral load.

= FL-ART: first-line AR.

According to the study criteria, 5.6% (3/41) of patients with TDR and fully active cART, 5.6% (1/18) of patients with TDR and non-fully active cART and 3.4% (9/264) of patients without TDR experienced virologic failure within 5 to 12 months after initiating first-line cART. The difference between the three groups was not significant in chi-square test (0.47) and logistic regression (TDR fully active cART vs. nonTDR: OR = 2.24, p = 0.24, TDR non-fully active cART vs. nonTDR: OR = .67, p = 0.64). There was also no significant difference in duration of first-line cART between the three groups (table 3). Two of the patients without TDR and none of the patients with TDR had transient viraemia with a VL between 500 and 1,000 copies/ml.

## Discussion

### Prevalence of Transmitted Drug Resistance

For the observation period from 1996 to 2010, an overall prevalence of 11.9% of TDR in Germany was identified in the HIV-1 Seroconverter Study, representing a mean slightly above the average reported from other European studies.

Comparable figures for the prevalence of TDR were reported in cohort studies from France (10.9%) [6], Spain (12.1%) [45], Italy (12.0%) [29], the UK (11.4% and 14.2%, respectively) [46], [47] and the Netherlands (13%) [7]. In contrast, other epidemiologic European studies reported levels of TDR prevalence below 10% [9], [13], [16], [21], [23], [26], [48].

Within the HIV-1 Seroconverter Cohort, time trends for TDR were rather stable. The two largest surveillance studies [20], [25] as well as other European cohorts [6], [26] also reported a stable prevalence of TDR. However, a stable trend was not observed in all European countries. Some countries report even declining prevalence of TDR [7], [10], [21], [24], [27]–[30].

The high but stable level of TDR which was observed in Germany might be influenced by different factors. In Germany the proportion of people newly infected with HIV but yet not diagnosed is estimated to be increasing [32], and onward transmission of resistant HIV among newly infected patients may occur. At the same time there is an increase in the proportion of HIV patients receiving antiretroviral therapy [32], and transmission of resistant HIV from treatment-experienced patients with unsuppressed viraemia may take place. Both events might contribute to the fact that TDR prevalence is stabilizing at a high level in Germany.

One factor which can also impact estimates of prevalence is the composition of the study population analysed: for HIV seroconverters a higher prevalence of TDR is reported than for patients with unknown duration of infection until resistance testing is performed [5], [19]. Reversal of fitness-impairing resistant mutations to the sensitive wild type during drug-naïve course of infection [25], [49], [50] may explain the lower prevalence of HIV resistance in patients with long-standing infections. Therefore, careful examination of the characteristics of study populations used to monitor TDR is required to compare the results of different studies.

The prevalence of TDR was in addition calculated with the Stanford algorithm to allow clinicians to put them into a clinical context. According to the Stanford algorithm the overall prevalence as well as the prevalence of NNRTI resistance, PI resistance, and dual-class resistance was higher than prevalence rates according to the SDRM list. Since according to the SDRM list substitutions resistance-associated positions in the viral enzymes are not considered which are observed to be polymorphic in some non-B subtypes (defined threshold value Bennett et al. 2009 [42]), the analysis of the predicted phenotypic resistance according to Stanford algorithm results as expected in overall higher prevalences of resistance in all drug classes.

### Resistance against Specific Drug Classes

In concurrence with other European studies, NRTI-associated resistance was most commonly found in the HIV-1 Seroconverter Cohort [6], [25]. The use of incomplete suppressive mono- and dual-NRTI regimen, facilitating the selection of resistant HIV, has declined since the early 2000s. Accordingly, a sizeable reduction in the prevalence of NRTI resistance was observed, in particular since the year 2000, resulting in a significant decrease in trend analysis. The reduced use of mono- and dual-NRTI treatment regimens, together with the lowered replication capacity of HIV harbouring fitness-reducing resistance mutations in the viral reverse transcriptase [51], [52], has already been suggested to contribute to the clear-cut decrease of NRTI resistance [39].

Trends in transmitted NNRTI and PI resistance were stable over time, extending and confirming previously published data describing the German situation [39]. In the case of PI resistance, this is most likely due to a higher genetic barrier of HIV to develop resistance to PI, in particular to ritonavir-boosted PIs. The accumulation of several resistance mutations is necessary to induce clinically relevant resistance [53]. In contrast to other studies, there is no increase of transmitted NNRTI resistance, which together with the low levels of PI resistance in newly infected patients may reflect a low proportion of drug resistance developed in patients under treatment and the high standard of care achieved in Germany.

The prevalence of transmitted dual (1.2%) and triple (0.3%) resistance was low at a total of 1.5%, which is also consistent with findings of other European studies [19], [22]. The relatively low occurrence of transmitted multi-resistant HIV is likely to reflect the low capacity for replication of the heavily mutated virus strains [54]. In accordance with findings from our previous [22], [39] and other studies [22], [25], [26], [55], subjects infected with HIV-1 subtype B had a significantly higher risk to contract a resistant virus in the case of a new infection in comparison to patients with HIV-1 non-B infections. This is most likely due to the widespread use of antiretroviral drugs in Europe and North America, especially for patients with a HIV-1 subtype B infection.

### Prescription Practice

For the majority of patients enrolled in the HIV-1 Seroconverter Cohort the prescription practice is in line with current international treatment guidelines which recommend that patients without TDR should receive two NRTIs and one NNRTI, or alternatively a boosted PI [56], [57]. Furthermore, prescription practices for individual drugs were related to treatment guidelines in most of the cases [57]–[59].

### Therapeutic Success

In our study there was no significant difference in the frequency of virologic failure between patients with and without TDR. he baseline parameters were comparable for both groups [60].

A sensitivity analysis was performed to compare the subpopulation that was included to assess therapeutic success with the patients of the cohort not included in analysis. The analysis revealed that the CD4-cell count/of patients included in the subpopulation was significantly lower than in excluded patients, both at the date of HIV-1 seroconversion and at the beginning of first-line cART277 vs. 329). Furthermore – and in line with previous findings – the time span between HIV-1 seroconversion and commencement of first-line cART was significantly shorter in the subpopulation (627 vs. 1,220 days). At the same time, the duration of first-line therapy was significantly higher in the subpopulation (1,954 vs. 244 days). This may be due to the fact that the inclusion criteria for selecting the subpopulation reduced the censoring for loss to follow up in the sample. It is possible that the low CD4-cell count at base line among the patients of the subpopulation triggered a more thorough monitoring of these patients, which in turn generated sufficient data to include them in the treatment response analysis in the first place. A number of studies could show that, at least over a shorter observational period, therapeutic effects for patients with TDR were comparable to those of patients without TDR when treated with a fully active regimen [8], [15], [37], [55]. However, these observations have been challenged, as some available data suggests that patients with TDR achieve worse levels of viral suppression throughout first-line cART, even when the drug regime is adjusted for existing resistance [34], [38]. According to the same study, it also took patients with TDR significantly longer to achieve viral suppression.

A possible cause for treatment failure despite adequate antiretroviral therapy is discussed for the presence of drug-resistant minor variants in the infected individual. Such resistant minorities are not detected by standard resistance testing based on population sequencing which was used in this study. Resistant viral strains in the viral quasispecies are only detected by population sequencing if they occur at least at a proportion of approximately 25% in the viral quasispecies. Mutations with very low prevalence down to 0.1% can be detected with ultrasensitive sequencing methods (ultra-deep sequencing); however, these tests are not routinely applied in resistance testing [61]. When these minorities were taken into account the estimated prevalence of TDR was clearly higher [55], [62]–[65], usually twice as high [55], [62], [63]. The clinical impact of such resistant minorities has not yet been conclusively established.

A number of studies have shown that treatment-relevant minor mutations led to a significant increase in therapeutic failure, especially in the case of NNRTI-containing regimen [61]–[63], [66], [67]. Other studies, however, did not report any effect of resistant minorities on either therapeutic success [55], [64] or immunologic parameters [64]. undetected minor resistant viral strains might have also contributed to virologic failure in groups.

### Use of Genotypic Resistance Test Results among Whole Study Population

The duration of first-line cART for all patients with TDR (198/1,667) included in the study was significantly shorter than it was for patients without TDR. Of the 123 patients with TDR, who also commenced first-line ART, 38.2% were not treated appropriately despite genotypic resistance results. One possible reason for the difference in duration of first-line treatment may thus be changes of the initial cART regime, if the latter proved inefficient.

There are numerous reasons for the high proportion of therapeutic regimes of the study population that were inappropriate in light of existing drug resistance. For more than half of the patients of the study population, treatment was initiated after 2004, which means that the lack of standardised testing for resistance which was true for the late 1990s and early 2000s cannot account for all of the observed results. Furthermore, a physician’s decision about the appropriate way to treat a resistant infection might not only focus on effectiveness but may also be influenced by the management of side effects and appropriate dosage of drugs. This may result in a compromise in that a not fully potent therapy is implicitly accepted.

### Limitations

Similar to other cohort studies with target populations of acute HIV-1 seroconverters, the proportion of male patients with a homosexual transmission risk is very high. Compared to German national HIV-registration data the proportion of MSM in the HIV-1 Seroconverter Study is clearly higher (51.3% vs. 87.9%). For this transmission group, which accounts for the largest share of HIV-infected patients in Germany [32], the data is representative. Conversely, a selection bias towards patients who get tested for HIV more frequently cannot be ruled out. Consequently, the results reported in this paper may not be representative for other demographic parameters and geographic regions.

A further limitation of this study is the need to define the threshold for the detection of VL at 500 copies/ml because of the long study period from 1996 to 2010, even though current VL assays are much more sensitive. It should be also noted, that the HIV-1 Seroconverter Study is an observational study. The lack of systematic recordings of data which are mandatory for the enrolment of patients in the treatment success analysis as well as data regarding changes of medication and compliance of patients makes it difficult to increase the sample size and establish causal relationships for observed differences within the cohort.

With regard to prevalence estimates of TDR, an underestimation of the real proportion cannot be ruled out. First, there was no testing for resistant minorities. Secondly, for documented HIV-1 seroconverters a period of three years could pass between the last negative and first positive HIV test. Because of this, reversions from resistant viruses to the sensitive wild type and, consequently, a reduction in the number of detectable mutations may have occurred. However, for 72.4% of the study population, the time span between both tests was less than a year, which distinctly limits the impact of potential reversions on the reported findings.

In conclusion, the prevalence of TDR within the HIV-1 Seroconverter Cohort was high at an overall level of 11.9% (1996–2010) and stable over time, which is consistent with findings of other European studies. The persistence of TDR in this and in other cohorts, as well as the increasing global migration of HIV-infected persons, underline the need for a comprehensive surveillance system for TDR in Germany.

For the majority of patients, the administered cART met current standards and complied with national and international treatment guidelines. Nevertheless, according to our findings, 38.2% of patients with TDR did not receive treatment that was adjusted for existing HIV resistances, which might explain the significant difference in the duration of treatment between patients with and without TDR.

Future investigations are needed to identify HIV minorities in plasma samples in order to analyse differences in outcome of first-line ART more deeply.
